# Indiana State Dental Association

**Published:** 1867-06

**Authors:** 


### INDIANA STATE DENTAL ASSOCIATION.

The 9th annual meeting of this Association will be held in INDIANAPOLIS, on TUESDAY, JUNE 25th, 1867, at 2 P. M. Ample arrangements have been made for the occasion, and it is confidently believed that the progressive spirit of the Dentists of our State will insure for the meeting a complete success.

Prof. Taft says: "I will endeavor to be at your State meeting in June," and other distinguished members of our profession are expected to visit us, and contribute their counsels of accumulated wisdom and experience.

The next meeting of the "American Dental Association" will be held in Cincinnati, and a full delegation from Indiana is desirable; but as this is a representative body, it is necessary to attend our State meeting in order to become delegates and participate in its deliberations. All Dentists in good standing are cordially invited to meet with us, and it is hoped that every Dentist in the State, who esteems his profession, will be present if possible.

PROGRAMME.

1st. The regular order of business, as prescribed by the Constitution and By-Laws.

2d. Clinics will be be had daily during the meeting.

3d. Miscellaneous business, to be called up at any time not assigned.

4th. The Microscope and its uses.

5th. "Dental Medicines."

6th. "Dental Anæsthetics."

7th. The "Bill to regulate the practice of Dentistry."

8th. A Library and Museum for the State Association.

9th. The Goodyear and Cummings Patents.

E. M. MORRISON, Noblesville,
*Chairman of Executive Com.*

J. KNAPP, *Pres't.*

FORT WAYNE.

J. F. JOHNSON, *Sec'y.*
INDIANAPOLIS.

It being difficult to engage a suitable room so far in advance of the time it will be wanted, as proposed by the Chairman of Executive Committee, the Secretary would suggest that Dentists call at Messrs. Strong & Smith's Dental Depot, South-West corner of Pennsylvania and Market Streets, where they will be informed of the place of meeting. It is but just to say to the profession, that the above named firm have kindly offered to furnish the operating chairs, and other appliances that may be required for clinical purposes by the Association, free of charge.

JOHN F. JOHNSTON *Secretary.*

INDIANAPOLIS, *March 25th*, 1867.

